# The clinical significance of integrin subunit alpha V in cancers: from small cell lung carcinoma to pan-cancer

**DOI:** 10.1186/s12890-022-02095-8

**Published:** 2022-08-04

**Authors:** Yu-Lu Tang, Guo-Sheng Li, Dong-Ming Li, Deng Tang, Jie-Zhuang Huang, Hao Feng, Rong-Quan He, Zhi-Guang Huang, Yi-Wu Dang, Jin-Liang Kong, Ting-Qing Gan, Hua-Fu Zhou, Jing-Jing Zeng, Gang Chen

**Affiliations:** 1 Department of Pathology, The First Affiliated Hospital of Guangxi Medical University, Nanning, Guangxi Zhuang Autonomous Region People’s Republic of China; 2 Department of Cardiothoracic Surgery, The First Affiliated Hospital of Guangxi Medical University, Nanning, Guangxi Zhuang Autonomous Region People’s Republic of China; 3 Department of Medical Oncology, The First Affiliated Hospital of Guangxi Medical University, Nanning, Guangxi Zhuang Autonomous Region People’s Republic of China; 4 Ward of Pulmonary and Critical Care Medicine, Department of Respiratory Medicine, The First Affiliated Hospital of Guangxi Medical University, Nanning, Guangxi Zhuang Autonomous Region People’s Republic of China; 5 Department of Medical Oncology, The Second Affiliated Hospital of Guangxi Medical University, Nanning, Guangxi Zhuang Autonomous Region People’s Republic of China

**Keywords:** Small cell lung cancer, Integrin subunit alpha V, Standardized mean difference, Area under the curve, Prognosis

## Abstract

**Background:**

Little is known about the relationship between integrin subunit alpha V (*ITGAV*) and cancers, including small cell lung cancer (SCLC).

**Methods:**

Using large sample size from multiple sources, the clinical roles of *ITGAV* expression in SCLC were explored using differential expression analysis, receiver operating characteristic curves, Kaplan–Meier curves, etc.

**Results:**

Decreased mRNA (SMD = − 1.05) and increased protein levels of ITGAV were detected in SCLC (*n* = 865). Transcription factors—ZEB2, IK2F1, and EGR2—may regulate *ITGAV* expression in SCLC, as they had ChIP-Seq (chromatin immunoprecipitation followed by sequencing) peaks upstream of the transcription start site of *ITGAV*. *ITGAV* expression made it feasible to distinguish SCLC from non-SCLC (AUC = 0.88, sensitivity = 0.78, specificity = 0.84), and represented a risk role in the prognosis of SCLC (*p* < 0.05). *ITGAV* may play a role in cancers by influencing several immunity-related signaling pathways and immune cells. Further, the extensive pan-cancer analysis verified the differential expression of *ITGAV* and its clinical significance in multiple cancers.

**Conclusion:**

*ITGAV* served as a potential marker for prognosis and identification of cancers including SCLC.

**Supplementary Information:**

The online version contains supplementary material available at 10.1186/s12890-022-02095-8.

## Introduction

Lung cancer is one of the most common cancers. Past research predicted that there would be more than 2,200,000 newly diagnosed lung cancer patients in 2020 worldwide, accounting for more than 10% of cancer cases [1, 2]. With an estimated 1,800,000 deaths in 2020, lung cancer is the leading cause of cancer death [1, 2]. Although several clinical management approaches have been applied, including surgery, chemotherapy, and molecular targeted therapy, the 5-year survival rate of lung cancer is still just 10–20% [2]. Nearly 13–15% of lung cancer cases are small cell lung cancer (SCLC) [3], whose 2-year survival has remained nearly unchanged (from 14% in 2009 to 15% in 2014) in America [1]. Thus, there is an urgent need to identify possible markers for distinguishing and treating SCLC.

SCLC is characterized by rapid growth and early metastasis [4, 5], and exploring markers from this perspective is likely to be a feasible strategy. Integrins regulate the localization and activity of proteolytic enzymes that promote cancer cell invasion and migration during tumor progression and metastasis. Integrin subunit alpha V (*ITGAV*) belongs to the integrin alpha chain family, and its encoded product is one component of integrins, which can bind with five integrin β subunits (β1, β3, β5, β6, and β8) [6, 7]. Data from several studies suggest that *ITGAV* is highly expressed in several cancers and demonstrates risk roles in the proliferation and migration of cancer cells [8, 9]. Moreover, the significant association of *ITGAV* expression and immune infiltrating cells (e.g., CD8+T cell and neutrophil) in certain cancers (e.g., colon cancer [10] and gastric cancer [11]) has been reported before, implying its potential in immunotherapy. However, no report on *ITGAV* and SCLC has been found in the literature, which makes it worth researching.

Based on many public and in-house samples, and for the first time, this study attempts to explore the expression, clinical significance, and underlying mechanisms of *ITGAV* in SCLC. With regard to conspicuous clinical values of *ITGAV* in SCLC, extensive pan-cancer analysis was also performed, contributing to understanding pathology mechanisms and potential application value of *ITGAV* in cancers.

## Materials and methods

The study was approved by the Ethics Committee of the First Affiliated Hospital of Guangxi Medical University, China.

### Collection of samples and performance of immunohistochemistry

Datasets (microarrays and RNA sequencing) for SCLC-related analyses were screened in several public databases, including ArrayExpress, Oncomine, Gene Expression Omnibus, and the GDC Data Portal. Strategies for searching datasets were: “(lung or bronch*) and (small cell) and (mRNA or gene).” The included criteria for datasets were: (1) *homo sapiens*-related cohort; (2) lung/bronchus tissues or cells; and (3) mRNA expression profile including *ITGAV* expression. The exclusion criteria were: (1) duplicate and/or incomplete expression profile, and (2) less than three samples in a combined dataset.

Clinical samples were collected and sliced for immunohistochemical detection of the ITGAV protein at the First Affiliated Hospital of Guangxi Medical University. Then, in-house samples were used to perform immunohistochemistry. The antibody—rabbit monoclonal to ITGAV (EPR16800)—applied in the study was purchased from Abcam plc. Immunohistochemical experiments were carried out according to the instructions of the manufacturer. Experimental methods and protein level scoring criteria can be found in our previous study [12].

For pan-cancer analyses, a dataset and its associated clinical information from the Xena database (developed by the University of California, Santa Cruz) was utilized in the study. Six types of samples (normal tissue, normal solid tissue, primary tumor, primary solid tumor, bone marrow, and primary blood derived cancer) were included for further research. Abbreviations of cancers involved in the pan-cancer dataset are listed in Additional file 1.

### Data standardization and elimination of batch effects between various datasets

All mRNA expression-associated data were transformed by log_2_ (*x* + 1). Datasets with the same platform (e.g., GSE32036 and GSE4127 from GPL6884) were merged for a combined dataset after eliminating batch effects [13–16], which was similar to the published study [12].

### The expression of *ITGAV* in SCLC

*Wilcoxon* rank-sum tests and a pooled standardized mean difference (SMD) were applied to assess the differential *ITGAV* expression between the SCLC group and the non-SCLC control group. A random effect model was used when there was significant heterogeneity between the datasets, which was judged by an *I*^2^ value of > 50% and/or a chi-square test *p* value of < 0.1. Otherwise, a fixed effect model was used for the SMD calculation. The SMD value had statistical significance only when its 95% confidence interval excluded zero. A *Begg*’s test was used to detect whether there was obvious publication bias (evaluated by *p* > 0.1) for the SMD analysis.

### The clinical significance of *ITGAV* expression in SCLC

*Wilcoxon* rank-sum tests were applied to identify whether *ITGAV* expression was relevant to the clinical features of SCLC patients. Kaplan–Meier curves and univariate Cox analysis were used to detect prognosis values of *ITGAV* expression in SCLC. In Kaplan–Meier curves, the optimal cut-point (using survminer software package) was used to classify high-*ITGAV* and low-*ITGAV* expressions. To confirm whether there was a significant correlation between prognosis and patient background, Fisher’s exact test was used to explore the age and TNM stage distribution differences between the high-*ITGAV* expression group and the low-*ITGAV* expression group, while Wilcoxon rank-sum test was also utilized to detect *ITGAV* expression levels in SCLC patients with various age and TNM stages.

Receiver operating characteristic curves (ROCs) and a summary ROC were used to detect the ability of *ITGAV* expression in distinguishing SCLC from non-SCLC, which was judged by the area under the curve (AUC) values (ranging from 0 to 1).

### The underlying mechanisms resulting in low-*ITGAV* expression in SCLC

A gene with a absolute value of log_2_ (fold change) ≥ 1 through the limma package [17–19] and SMD < 0 was identified as having low-expression genes (LEGs) in SCLC. A gene that was positively related to *ITGAV* expression (Pearson coefficient ≥ 0.3, *p* < 0.05) in > 30% (4/12) datasets was considered as *ITGAV*-positively-related genes (*ITGAV*-PRGs). Based on chromatin immunoprecipitation followed by sequencing (ChIP-Seq) data, the Cistrome data browser was feasible to predict transcription factors (TFs) possibly regulating *ITGAV* expression. A TF encoded by a gene with triple identity—LEG, *ITGAV*-PRG, and predict TF—was considered a potential TF regulating *ITGAV* expression.

### The potential mechanisms of *ITGAV* in SCLC

Based on *ITGAV*-related LEGs, underlying mechanisms of *ITGAV* expression in SCLC were explored using ontology terms and signaling pathways. Ontology terms in the study included disease ontology and gene ontology, while pathways were from the Kyoto Encyclopedia of Genes and Genomes (KEGG) [20] and Reactome. Gene set enrichment analysis (GSEA) for GO and KEGG was also conducted to verify findings from term and pathway analyses.

To detect associations between *ITGAV* expression and tumor immune microenvironment (TME), the relationship between *ITGAV* expression and immune infiltration levels (using Pearson coefficient) was explored based on the ESTIMATE algorithm [19, 21–23] and CIBERSORT algorithm [14, 24, 25]. Immune checkpoints were promising in the treatment of cancers [26], and thus, the study also evaluated their associations with *ITGAV* expression to preliminarily assess the immunotherapy potential of *ITGAV* in SCLC.

### Pan-cancer analyses

The SMD was applied in SCLC-related rather than pan-cancer-associated calculations, as there was just one dataset for each cancer in the pan-cancer dataset, and it was not suitable for performing SMD evaluation. Except for SMD, TF prediction, and enrichment analyses, statistical methods used in pan-cancer analyses were the same as in research on SCLC. All data for pan-cancer analyses are shown in Additional file 2.

### Statistical analysis

In this study, *p* < 0.05 indicates statistical significance unless there was an additional explanation. Violin plots, forest plots, Kaplan–Meier curves, ROCs, box plots, GO plots, and heatmaps were generated by a series of Bioconductor packages [27, 28] in R software (v4.1.0).

## Results

### Overview of samples included in the study

For SCLC, 28 datasets from public databases were finally selected and classified into 12 combined datasets based on the same platforms. The 12 datasets contained 363 SCLC samples and 532 non-SCLC control samples. Also, 26 SCLC and 29 non-SCLC in-house samples were included for exploring ITGAV protein levels in SCLC. Thus, 1022 samples (*n* of SCLC = 389, *n* of non-SCLC = 561) were eventually selected for research of ITGAV on SCLC. Selection processes and details of SCLC-related samples can be seen in Additional files 3 and 4.

In pan-cancer analyses, *ITGAV* expression in 26 cancers (with not less than three samples for each cancer) was explored, involving 21,989 samples (*n* of cancer = 11,537; *n* of non-cancer = 10,452). Then, 12,106 samples (in 39 cancers) with overall survival information and 5666 samples (in 32 cancers) with disease-free interval data were utilized for survival analyses (Additional file 2).

### ITGAV expression in SCLC and non-SCLC

**Fig. 1:**
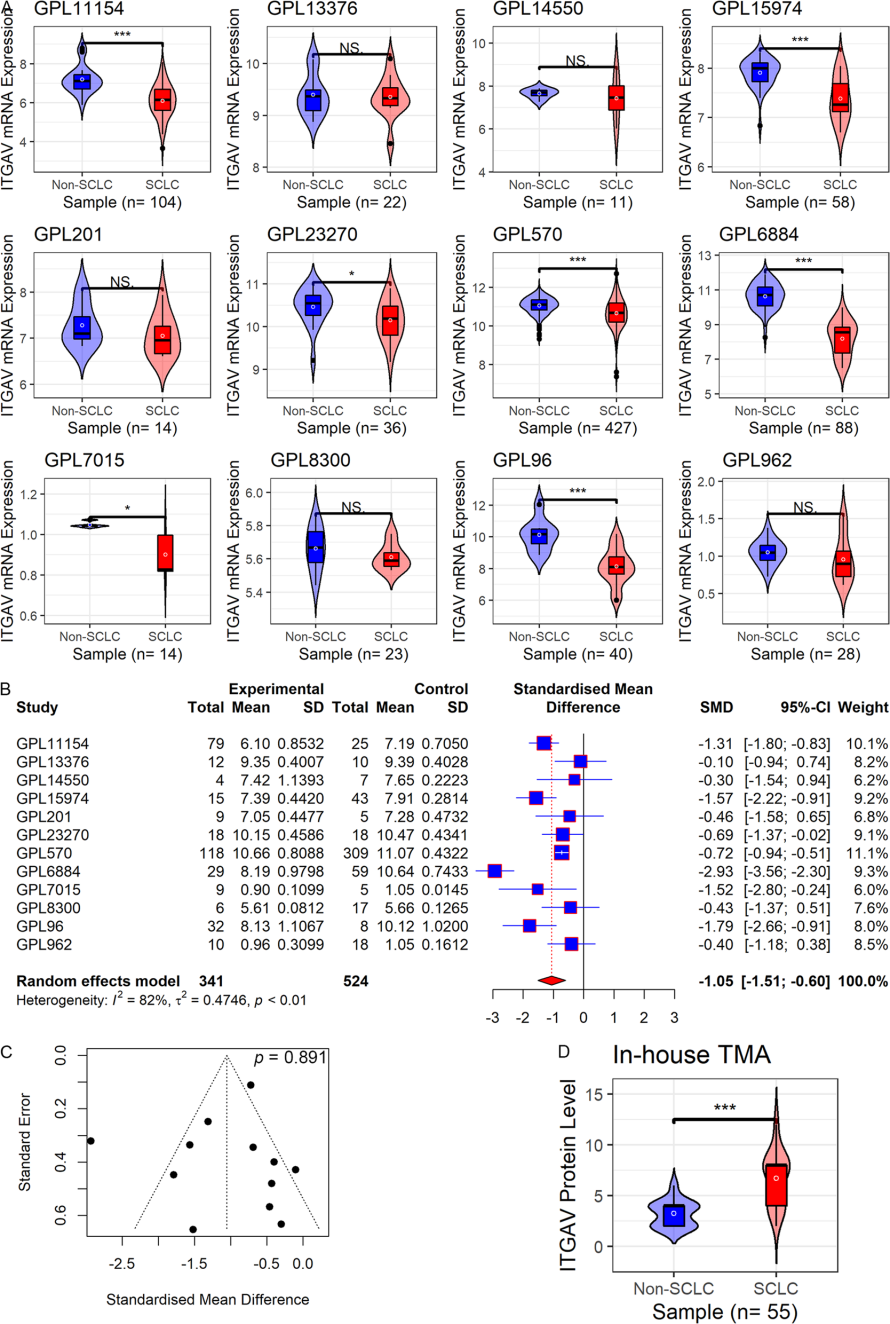
The expression of ITGAV in small cell lung carcinoma (SCLC). Panel **A** Violin plots of ITGAV expression in SCLC. Panel **B** A forest plot of evaluating standard mean difference (SMD) of ITGAV expression between SCLC and non-SCLC groups. Panel **C** A funnel plot with Begg’s test for publication bias test. Panel **D** A violin plot of ITGAV protein levels between SCLC and non-SCLC groups. *TMA* tissue microarray

Compared to the non-SCLC group, *ITGAV* mRNA expression was downregulated in the SCLC group, which was reported by 7/12 datasets included in the study (*p* < 0.05, Fig. 1A). The consistent conclusion was also sustained by the random effects model (SMD =  − 1.05, 95% CI did not contain 0, Fig. 1B), and no significant publication bias was detected for SMD results (*Begg’s* test, *p* = 0.891, Fig. 1C).

**Fig. 2:**
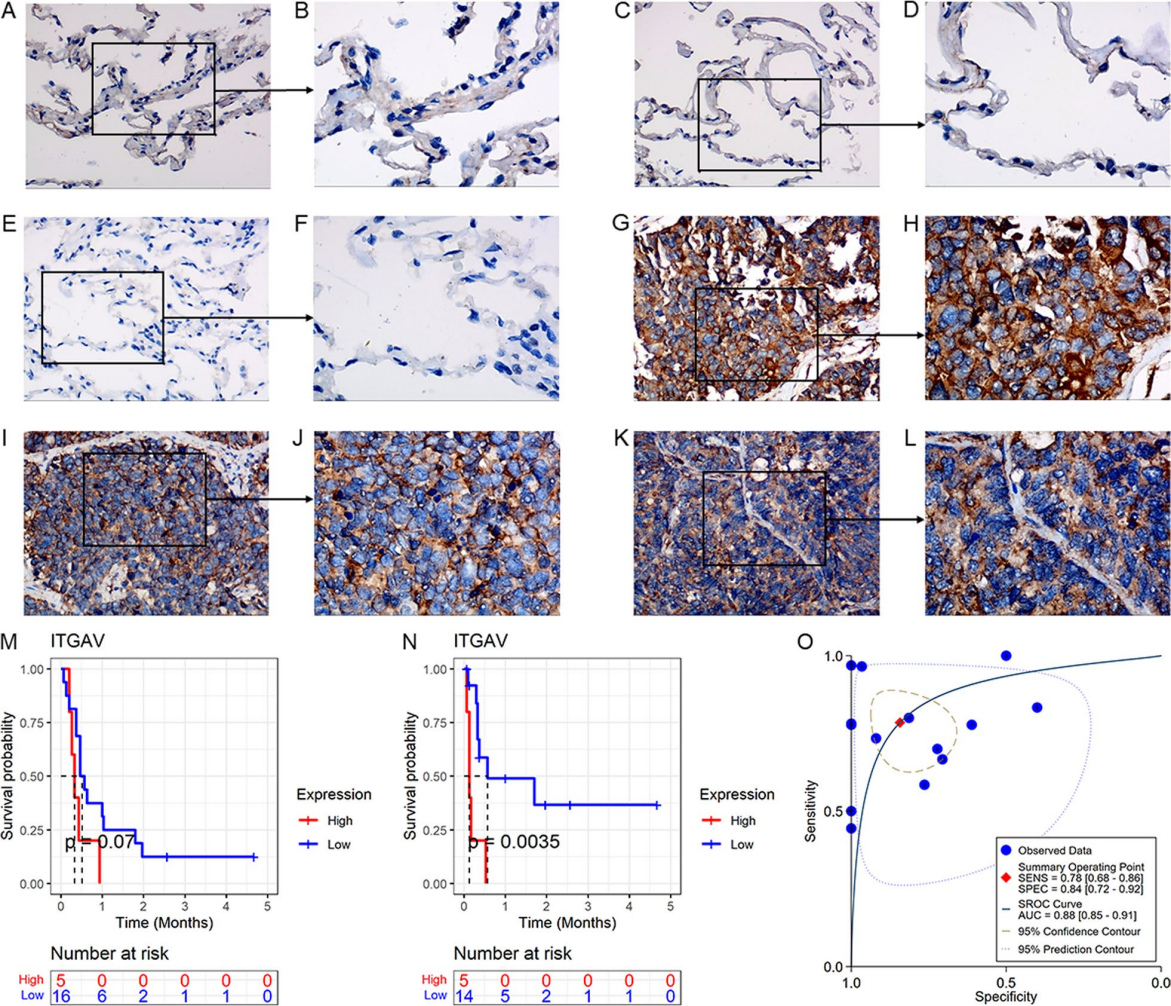
ITGAV protein levels, and the clinical significance of ITGAV expression in small cell lung carcinoma (SCLC). Panels **A**–**L** The protein levels of ITGAV in non-SCLC (**A**–**F**) and SCLC (**G**–**L**) tissues under the microscope by in-house tissue microarrays. The left figure of each two combined figures is 200×, and the right figure is 400×. Panel **M** A Kaplan–Meier curve of overall survival between high- and low-ITGAV mRNA expression groups. Panel **N** A Kaplan–Meier curve of disease-free survival between high- and low-ITGAV mRNA expression groups. Panel **O** Summary receiver operating characteristic curve for identifying SCLC based on ITGAV mRNA expression. *SENS* sensitivity, *SPEC* specificity, *AUC* area under the curve

At the protein level, positive ITGAV expression was detected in SCLC tissues rather than non-SCLC tissues (Figs. 2A–L). As a result, higher ITGAV protein levels were found in SCLC tissues (*p* < 0.001, Fig. 1D).

### The clinical significance of *ITGAV* expression in SCLC

#### The correlation between *ITGAV* expression and clinical parameters

No statistical correlation between *ITGAV* expression and TNM stage, clinical stage, or age was found in SCLC (data not shown), indicating that the expression of *ITGAV* in SCLC was likely not affected by these factors and that *ITGAV* was a factor independent of these indicators.

SCLC is related to smoking history [29, 30], and thus the relationship between *ITGAV* expression and smoking history was investigated in this study. As shown in Additional file 5, SCLC patients with high-*ITGAV* expression had a more extended smoking history.

#### The prognosis values of *ITGAV* expression in SCLC

As seen in Figs. 2M–N, high-*ITGAV* mRNA expression was associated with unfavorable overall survival and disease-free survival (i.e., the period between the surgery date and the recurrence date), and the latter was statistically significant. For disease-free survival, there was no difference in age and TNM stage distribution between the high-*ITGAV* expression group and the low-*ITGAV* expression group (Additional file 6), and no *ITGAV* expression levels were detected in SCLC patients with various clinical parameters (Additional file 7). Thus, *ITGAV* expression represented an independent risk role in the prognosis (disease-free survival) of SCLC patients.

#### The identification effects of *ITGAV* expression in SCLC

With mRNA expression of 12 datasets (containing 895 samples), *ITGAV* demonstrated conspicuous effects in distinguishing SCLC from non-SCLC (AUC = 0.88, sensitivity = 0.78, specificity = 0.84; Fig. 2O), suggesting its potential in identifying SCLC.

### The underlying mechanisms resulting in low-*ITGAV* expression in SCLC

**Fig. 3:**
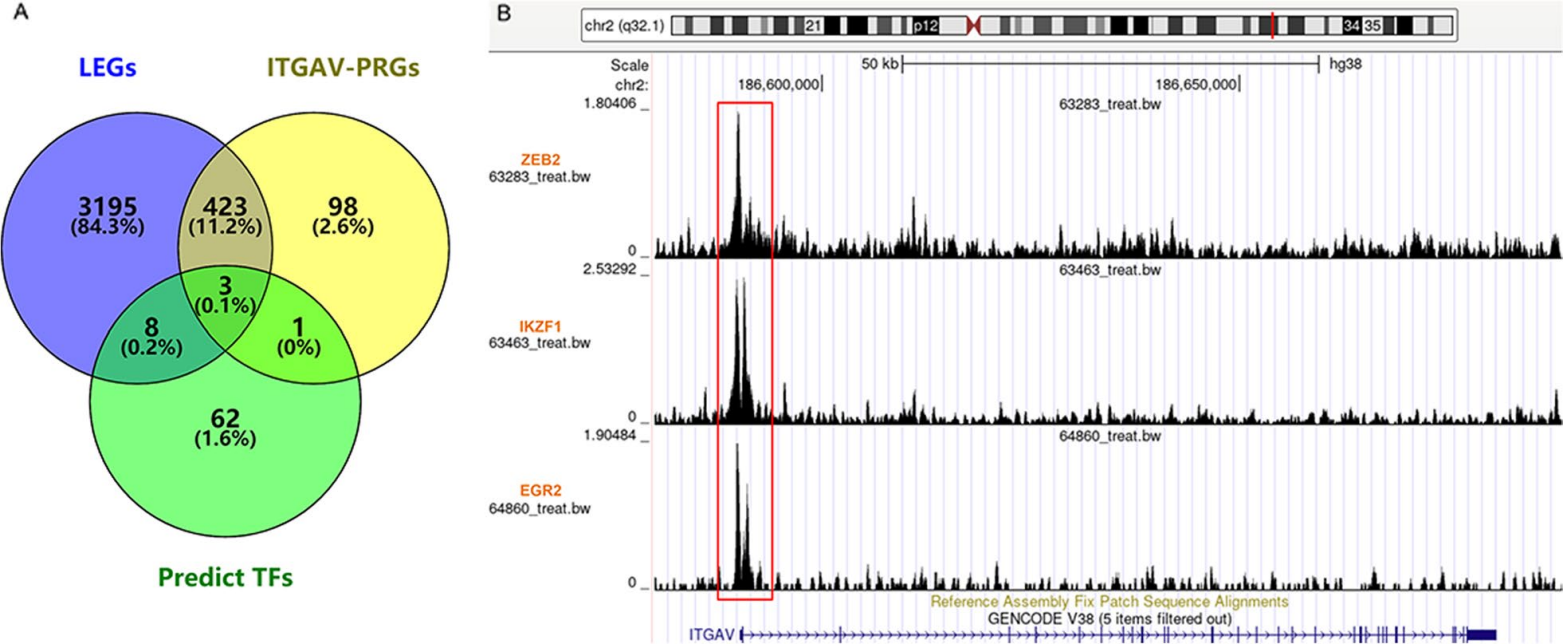
Identification of potential transcription factors regulating ITGAV expression. **A** The venn plot for screening predicted transcription factors for ITGAV. **B** For the three transcription factors, there existed binding sites with the potential promoter region of ITGAV

After calculation, 3629 LEGs were identified, with the absolute value of log_2_ (fold change) ≥ 1 and SMD < 1 (data not shown). Also, 525 genes were considered *ITGAV*-PRGs in > 30% (4/12) datasets (data not shown). In the Cistrome data browser, 74 predicted TFs likely regulated *ITGAV* expression, and 423 *ITGAV*-positively-related low-expression genes (*ITGAV*-PRLEGs) were merged from LEGs and *ITGAV*-PRGs. Through the intersection of LEGs, *ITGAV*-PRGs, and predicted TFs, three TFs—ZEB2, IK2F1, and EGR2—were screened (Fig. 3A). All three TFs were considered potential TFs regulating *ITGAV* expression, as they had ChIP-Seq peaks upstream of the transcription start site of *ITGAV* (Fig. 3B).

### Potential mechanisms of *ITGAV* in SCLC

#### Enrichment analyses of *ITGAV*-PRLEGs in SCLC

**Fig. 4:**
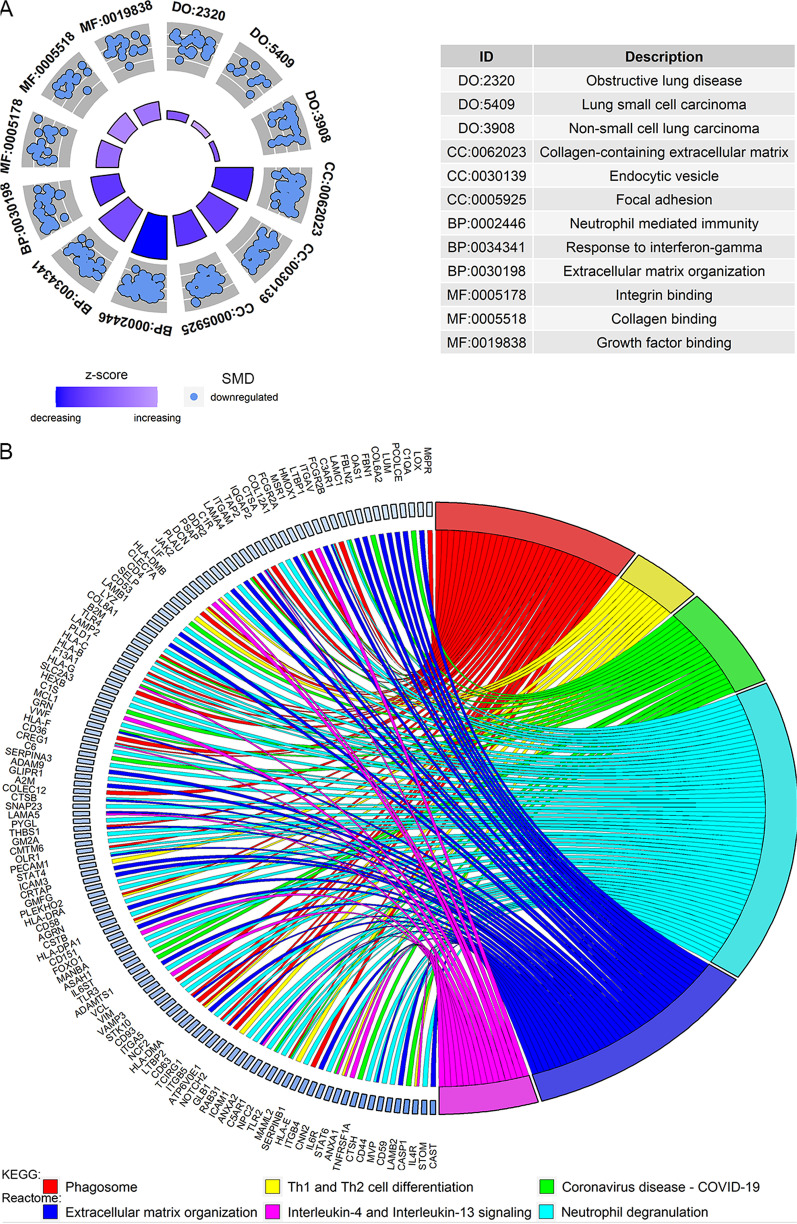
Disease ontology terms, gene ontology terms, kyoto encyclopedia of genes and genomes and reactome pathways of ITGAV-positively-related downregulated expression genes. *DO* disease ontology, *CC* cellular component, *BP* biological process, *MF* molecular function

Through disease ontology, *ITGAV*-PRLEGs were associated with lung diseases, small cell lung carcinoma, and non-small cell lung carcinoma (Fig. 4A). They tend to participate in the composition of “collagen-containing extracellular matrix,” “focal adhesion,” and “endocytic vesicle” (cell components); are involved in biological processes such as “response to interferon-gamma,” “neutrophil mediated immunity,” and “extracellular matrix organization”; and affect multiple molecular functions (e.g., “integrin binding,” “collagen binding,” and “growth factor binding”).

**Fig. 5:**
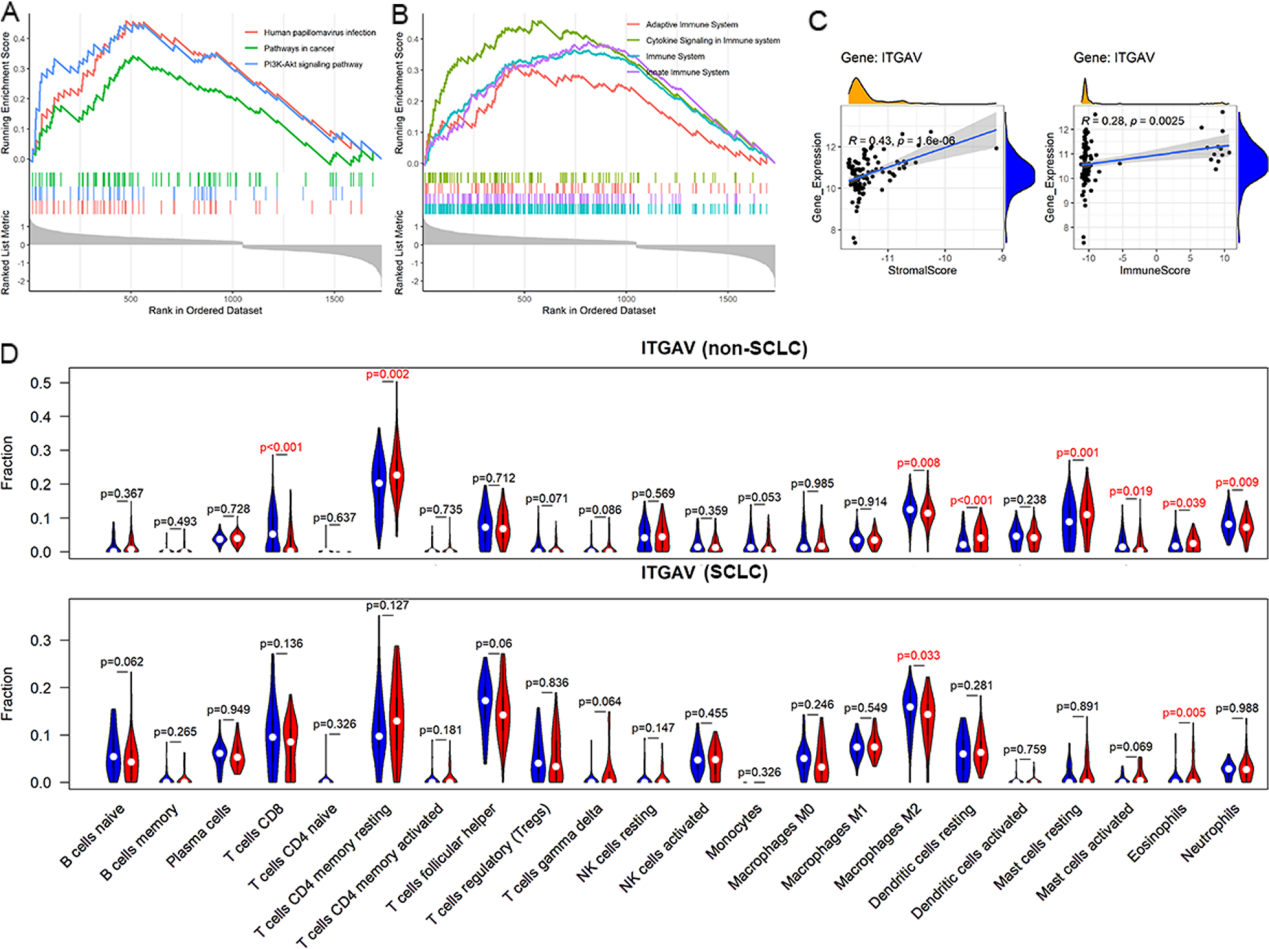
Gene set enrichment analysis (GSEA), and immune infiltration levels analyses. Panels **A** and **B** GSEA for Kyoto Encyclopedia of Genes and Genomes (panel **A**), and Reactome pathways (panel **B**) of ITGAV-positively-related downregulated expression genes. Panels **C** and **D** Analyses of immune infiltration levels based on ESTIMATE (panel **C**) and CIBERSORT (panel **D**) algorithms. *SCLC* small cell lung carcinoma

*ITGAV*-PRLEGs clustered in immune and virus-related KEGG pathways, some examples of which were “phagosome,” “Th1 and Th2 cell differentiation,” and “Coronavirus disease—COVID-19” (Fig. 4B). *ITGAV*-PRLEGs also aggregated in immune and ECM-associated Reactome pathways (Fig. 4B). Thus, a close relationship between *ITGAV*-PRLEGs and immunity can be seen. This finding was also supported by GSEA results (Fig. 5A, B).

#### Analysis of the relationship between *ITGAV* expression and TME

Based on the ESTIMATE algorithm, *ITGAV* expression in SCLC was positively related to stromal scores and immune scores (Fig. 5C). However, there was no statistical significance for ESTIMATE score and tumor purity (data not shown).

To further explore the relevance of *ITGAV* expression with immune cell infiltration levels, the CIBERSORT algorithm was applied. As a result, *ITGAV* expression was negatively related to activated immune infiltration levels of several immune cells (e.g., CD8 T cells, M2 macrophages, and active mast cells) and positively associated with resting immune cells (e.g., memory CD4 T cells and dendritic cells; Fig. 5D). However, such findings were seen in the non-SCLC group rather than the SCLC group, as no statistical significance was detected for the latter (Fig. 5D).

Taken together, *ITGAV* showed a close correlation with immune stroma score, immune score, and a few immune cells (i.e., M2 macrophage cells and eosinophils), implying its potential mechanisms in SCLC.

### *ITGAV* in pan-cancer

With regard to differently expressed *ITGAV* and its clinical significance in SCLC, we attempted to perform a similar exploration in pan-cancer analysis, which could contribute to the understanding and application of *ITGAV* in cancers.

#### The expression of *ITGAV* between SCLC and non-SCLC

**Fig. 6:**
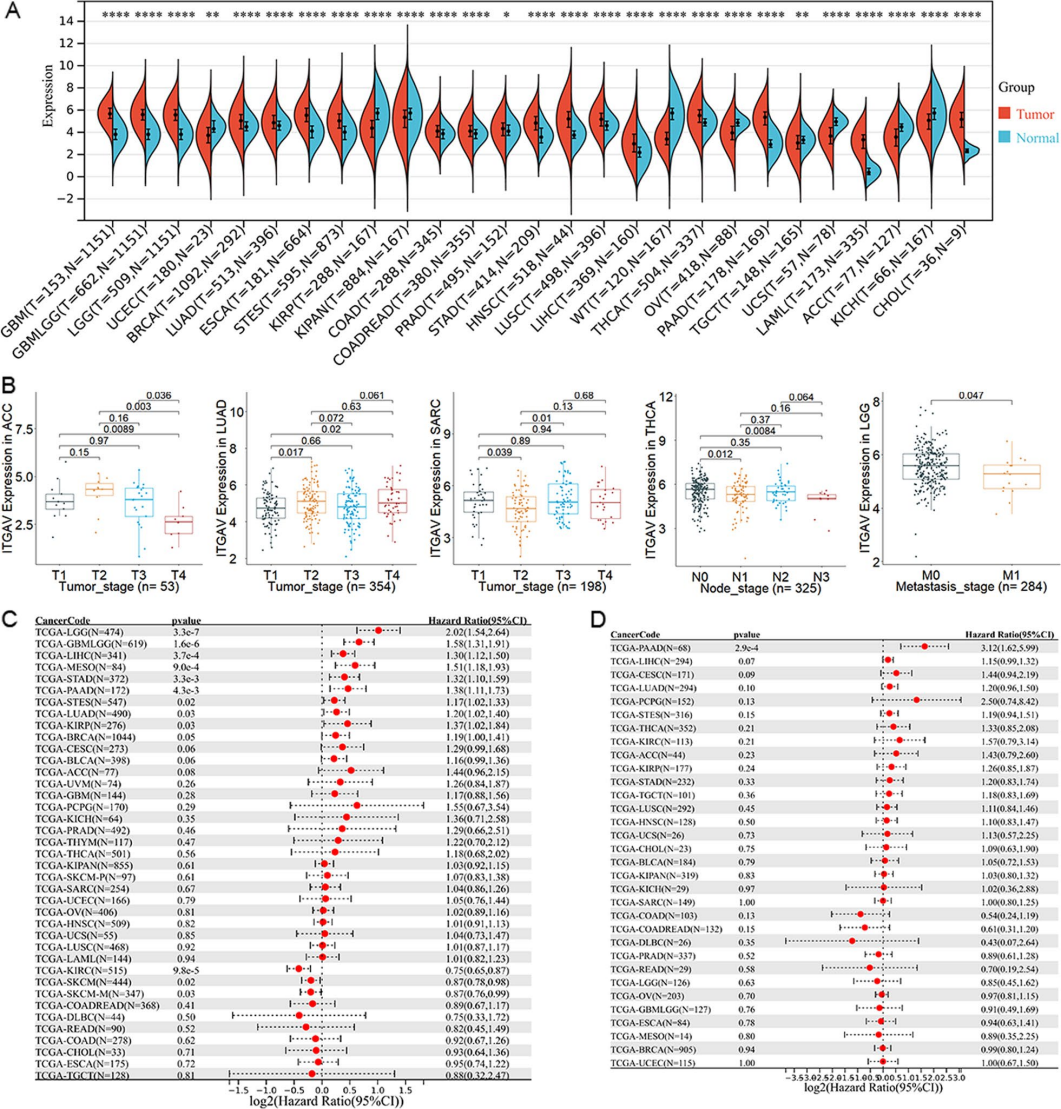
The expression of ITGAV, and its associations with clinical parameters and prognosis in pan-cancer. Panel **A** The expression of ITGAV mRNA expression in pan-cancers. *p < 0.05. Panel **B** Associations of ITGAV in clinical parameters in multiple cancers. Panel **C** The correlation of ITGAV expression with the overall survival of cancers patients. Panel **D** The correlation of ITGAV expression with the disease-free survival of cancers patients

Various expressions can be seen between cancer and non-cancer groups. Elevated *ITGAV* expression was found in 19 cancers, such as glioblastoma multiforme, glioma, and brain lower grade glioma (LGG; Fig. 6A). This phenomenon also existed in LUAD and LUSC (both belonged to NSCLC; Fig. 6A), contrary to SCLC. Eight cancers (e.g., uterine corpus endometrial carcinoma) were found with low-*ITGAV* expression in cancer groups rather than non-cancer groups (Fig. 6A).

#### The correlation between *ITGAV* expression and clinical parameters

For most of the 39 cancers, there was no statistical correlation between *ITGAV* expression and TNM stage or clinical stage, except for adrenocortical carcinoma, LUAD, sarcoma, thyroid carcinoma, and LGG (Fig. 6B). This revealed that *ITGAV* expression was an independent factor (not affected by TNM stage and clinical stage) for most cancers.

#### The clinical significance of *ITGAV* expression in pan-cancer

In the 39 cancers explored in the study, *ITGAV* expression was relevant to the poor overall survival of 9/39 cancers: LGG, glioma, liver hepatocellular carcinoma (LIHC), mesothelioma, stomach adenocarcinoma, pancreatic adenocarcinoma (PAAD), stomach, esophageal carcinoma, LUAD, and kidney renal papillary cell carcinoma (*p* < 0.05; Fig. 6C). That is, *ITGAV* expression consistently played risk roles in all nine cancers, indicating the risk factor of *ITGAV* for the overall survival of cancer patients. *ITGAV* expression was also identified as a risk factor for disease-free survival of PAAD (*p* < 0.05; Fig. 6D), although this was not seen in other cancers.

**Fig. 7:**
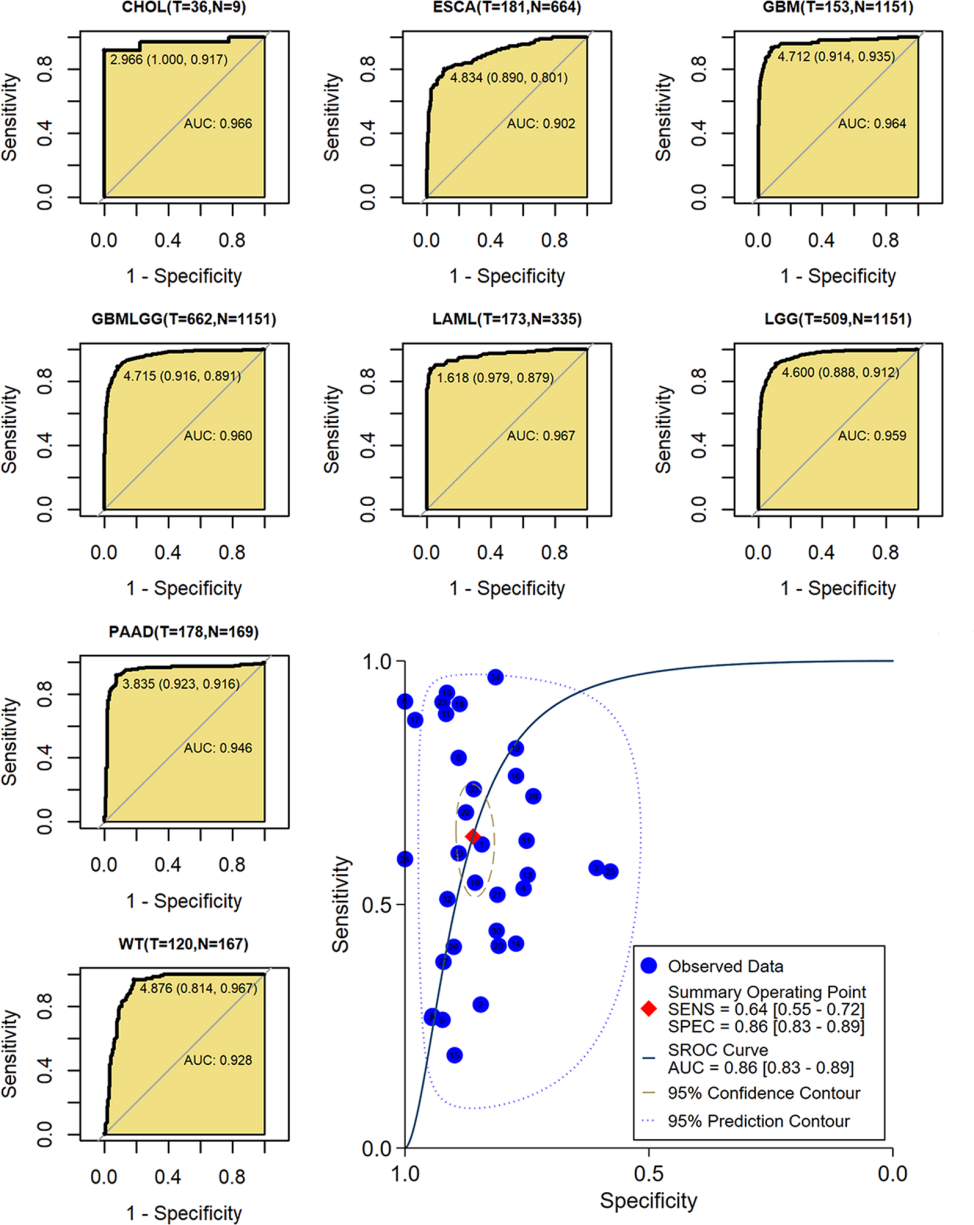
Receiver operating characteristic curves and a summary receiver operating characteristic curve for identifying cancers from non-cancers based on ITGAV expression. *SENS* sensitivity, *SPEC* specificity, *AUC* area under the curve

Similar to in SCLC, *ITGAV* expression showed significantly distinctive effects between cancers and non-cancers, particularly for cholangiocarcinoma, esophageal carcinoma, glioblastoma multiforme, glioma, acute myeloid leukemia, LGG, PAAD, and high-risk Wilms tumors (all AUCs > 0.9; Fig. 7). AUC values for other cancers can be seen in Additional file 8. In summary, the AUC of ROC for all 34 cancers was up to 0.86, suggesting that *ITGAV* expression has the potential to screen cancers (Fig. 7).

#### Immune-related analyses of *ITGAV* in pan-cancer

**Fig. 8:**
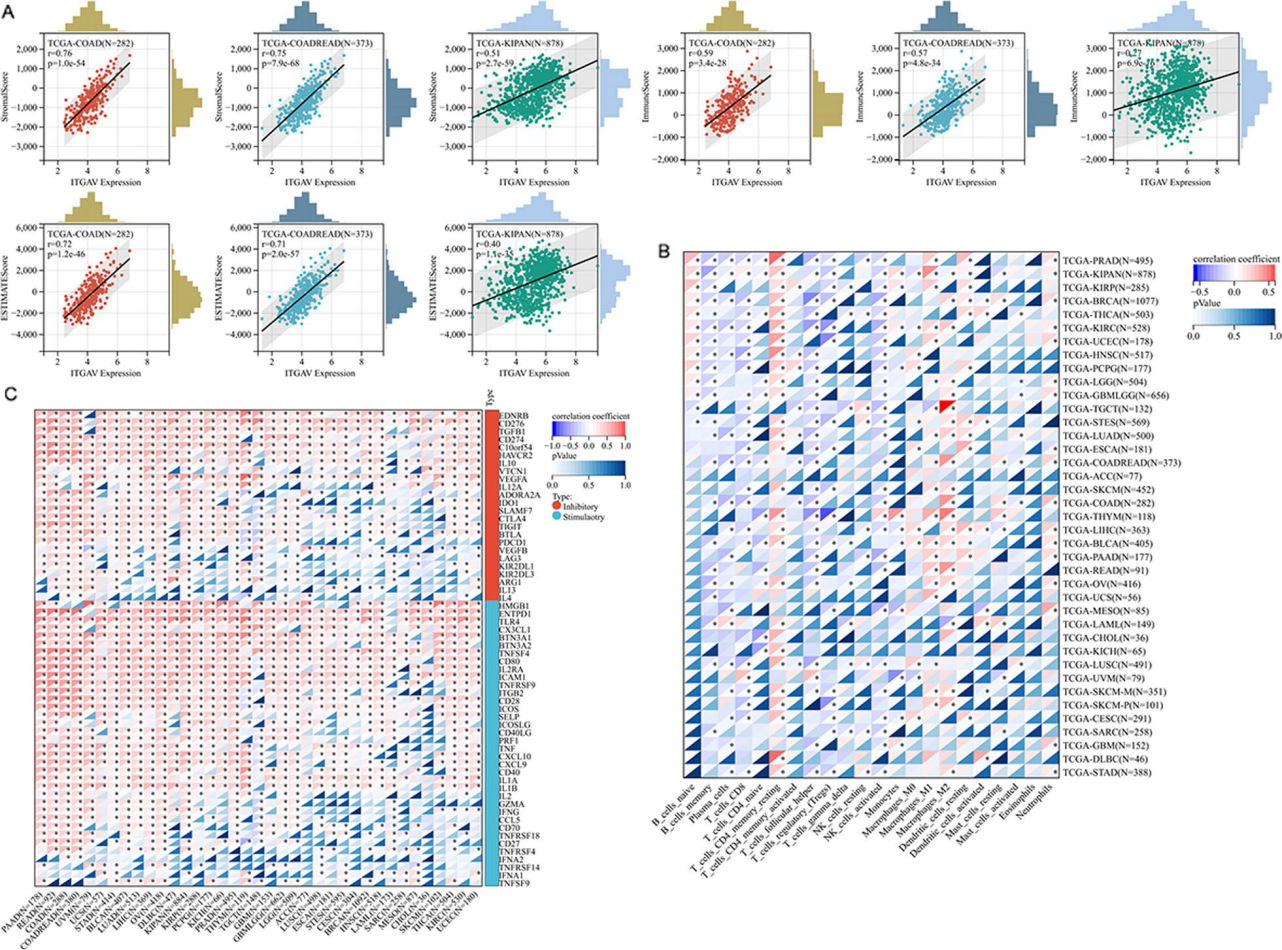
The association of ITGAV expression with immune infiltration level in pan-cancer

Through analyses of immune infiltration levels of pan-cancer, the *ITGAV* expression in 11 cancers was relevant to stromal score, immune score, and ESTIMATE score (Pearson correlation coefficient** > **0.2, *p* < 0.05; Table 1), and three examples of them were COAD, COADREAD, and KIPAN (Fig. 8A). Interestingly, no negative associations, but consistent positive relationships, were observed in the series of findings.

**Table 1:** Pearson correlation of ITGAV expression and immune infiltration levels in cancers

Cancers	Stromal score		Immune score		Estimate score	
	Correlation coefficient	*p*	Correlation coefficient	*p*	Correlation coefficient	*p*
GBM	0.239	0.003	0.275	0.001	0.269	0.001
GBMLGG	0.244	< 0.001	0.231	< 0.001	0.243	< 0.001
LGG	0.248	< 0.001	0.21	< 0.001	0.231	< 0.001
LUAD	0.413	< 0.001	0.207	< 0.001	0.325	< 0.001
COAD	0.762	< 0.001	0.593	< 0.001	0.722	< 0.001
COADREAD	0.747	< 0.001	0.574	< 0.001	0.705	< 0.001
KIPAN	0.51	< 0.001	0.268	< 0.001	0.403	< 0.001
READ	0.71	< 0.001	0.535	< 0.001	0.668	< 0.001
PAAD	0.573	< 0.001	0.339	< 0.001	0.479	< 0.001
OV	0.343	< 0.001	0.272	< 0.001	0.332	< 0.001
BLCA	0.404	< 0.001	0.264	< 0.001	0.356	< 0.001

*GBM* glioblastoma multiforme, *GBMLGG* glioma, *LGG* brain lower grade glioma, *LUAD* lung adenocarcinoma, *COAD* colon adenocarcinoma, *COADREAD* colon adenocarcinoma/rectum adenocarcinoma esophageal carcinoma, *KIPAN* pan-kidney cohort; rectum adenocarcinoma, *PAAD* pancreatic adenocarcinoma, *OV* ovarian serous cystadenocarcinoma, *BLCA* bladder urothelial carcinoma

Based on the CIBERSORT algorithm, the relevance between *ITGAV* expression and multiple immune cells is evident. Positive associations (Pearson correlation coefficient** > **0.2, *p* < 0.05) of *ITGAV* expression with resting CD4 memory T cells, M1 macrophages, and M2 macrophages were observed in at least ten cancers (Fig. 8B). At not less than ten cancers, negative relationships can be observed (Pearson correlation coefficient < − 0.2, *p* < 0.05) of *ITGAV* expression with memory B cells, CD8 T cells, follicular helper T cells, regulatory T cells, and activated NK cells (Fig. 8B). In addition, *ITGAV* expression was related to the expression of many immune checkpoints in a series of cancers (Fig. 8C) and revealed the immunotherapy potential of *ITGAV*.

## Discussion

Using large samples from multiple sources, this study comprehensively identified different *ITGAV* expression in SCLC and revealed the conspicuous prognosis and identification values of *ITGAV* expression in this disease. Several TFs that may regulate *ITGAV* expression in SCLC were also identified, which has not been reported. Focusing on TME, the study explored the molecular mechanisms of *ITGAV* in SCLC. Further, the extensive pan-cancer analysis verified the differential expression of ITGAV and its clinical significance in multiple cancers with tens of thousands of samples.

Differential expression of *ITGAV* in cancer is common, and its high expression is predominant. Upregulated *ITGAV* expression has been identified in multiple cancers, such as gastric cancer cells [7], breast cancer [8], and hepatocellular carcinoma [9]. Downregulated *ITGAV* expression has also been emphasized in epithelial ovarian cancer [31]. Thus, it is necessary to comprehensively explore *ITGAV* expression in many cancers. In this study, among 35 cancers (including SCLC), 80% (28/35) demonstrated different expression levels between cancer groups and non-cancer groups, and high expression was observed in 68% (19/28) of the latter. One point that should be noted was that low-*ITGAV* mRNA and high-ITGAV protein levels were detected. These seemingly inconsistent results were reasonable, since (1) polypeptides can be produced by an mRNA molecule, but their translation rates may vary in space and time; (2) mRNA molecules from the same gene may feasibly produce various amounts of protein due to translational heterogeneity [32]; (3) the protein product of a gene tended to be more stable than its mRNA, as the latter degrades more easily [33]. Therefore, inconsistent *ITGAV* mRNA and protein levels are still rational in some conditions.

Low-*ITGAV* mRNA expression in SCLC likely resulted from downregulated expression of three TFs—ZEB2, IK2F1, and EGR2. The three TFs may be regulators of *ITGAV* expression, as they demonstrated decreased expression and close positive relationships with *ITGAV* expression in SCLC and had ChIP-Seq peaks upstream of the transcription start site of *ITGAV*. Previous reports have demonstrated that some of the three TFs are essential factors in SCLC. For instance, Wang et al. [34] found that ZEB2 participated in promoting the occurrence of extracellular matrix in SCLC, thus contributing to the progression of the disease. However, to the best of our knowledge, no relevant research on the regulation of ZEB2, IK2F1, and EGR2 for *ITGAV* exists, which to some extent indicates the novelty of this study.

*ITGAV* expression demonstrated conspicuous clinical significance in quite a few cancers. The gene has been recognized as having a vital risk role in cancer progression. For example, Cheuk et al. [8] showed high-ITGAV expression causing breast cancer metastasis; Kemper et al. [35] identified that *ITGAV* expression contributed to PAAD; Loeser et al. [6] revealed the relationship between *ITGAV* expression and unfavorable prognosis for patients with esophageal adenocarcinoma. However, no similar research has been reported for SCLC. Although reduced *ITGAV* mRNA expression was detected in SCLC, we believe that the roles of *ITGAV* in SCLC resulted from its high protein levels for several reasons.

First, the function of a gene is usually due to its coding protein rather than its mRNA [32]. Second, its association with a poor prognosis of SCLC (found in the current study) supported *ITGAV* as a risk factor in SCLC. Further pan-cancer analyses demonstrated that *ITGAV* expression was related to the poor overall survival of patients with LGG, glioma, LIHC, mesothelioma, stomach adenocarcinoma, PAAD, stomach, and esophageal carcinoma, LUAD, and kidney renal papillary cell carcinoma; moreover, it was also associated with shorter disease-free survival of PAAD patients. Among these cancers, *ITGAV* has been identified as a risk factor in LIHC [9], mesothelioma [36], and PAAD [35], while no reports about the remaining six cancers indicated the novelty of our study. The finding that *ITGAV* expression makes it feasible to distinguish multiple cancer tissues from their controls suggests the potential of *ITGAV* expression in screening cancers. *ITGAV* may serve as an essential marker of prognosis and identification of multiple cancers.

Through disease ontology, *ITGAV*-PRLEGs are involved in some diseases, including SCLC and non-small-cell lung carcinoma, suggesting associations between *ITGAV* and lung cancers. For *ITGAV*-PRLEGs, the keywords for gene ontology were “adhesion,” “extracellular matrix,” and “immunity.” Cell–cell interactions and cell adhesion are key mediators of lung cancer progression, including immune evasion and metastatic events [37]. Integrins take part in cell surface adhesion and signaling and have essential functions in cancer progression. The protein product encoded by *ITGAV* is a subunit of integrins (the other is the β subunit), and thus participates in cancer development [6, 7]. For example, upregulated *ITGAV* stimulated the synergistic effect of integrin and selectin, which promoted adhesion between PAAD and peritoneal mesothelial cells, finally leading to the growth of pancreatic cancer [35]. It can be seen that the typicle role of *ITGAV* in cell adhesion may be one of its potential mechanisms in SCLC. Little is known about its immune-related role in cancers, although this finding was evident in this study.

The roles of *ITGAV* in cancers may be linked to TME. In our study, signaling pathways of KEGG, Reactome, and GSEA consistently demonstrated that *ITGAV*-PRLEGs were clustered in immune-related pathways. Moreover, *ITGAV* expression was positively associated with scores for all immune stromal cells, immune cells, and estimated scores (tumor purity), suggesting its close associations with TME. Of the multiple components of TME, immune cells were recognized as the predominant factors in regulating cancer progression [38]. Positive associations between *ITGAV* expression and resting CD4 memory T cells, M1 macrophages, and M2 macrophages were observed in more than ten cancers. Macrophages demonstrated a dual role in tumor progression. On one hand, based on proinflammatory cytokines and cytotoxic activities, they tend to inhibit tumor growth; on the other hand, they are more likely to stimulate tumor proliferation, angiogenesis, and metastasis, and as a result, trigger tumor progression [38]. Thus, a positive correlation between *ITGAV* expression and macrophage infiltration levels may support its relationship with poor prognosis in cancer patients.

More importantly, negative relationships were observed between ITGAV expression and infiltration levels of memory B cells, CD8 T cells, and activated NK cells. Interestingly, CD8 T cells and activated NK cells were prominent factors (notably CD8 T cells) in controlling tumor progression [39, 40]. Their negative relationship with *ITGAV* supported the risk roles *ITGAV* played in most cancers. Studies have shown that immune checkpoints are thought to be involved in immunosuppression, thereby preventing immune cells from eliminating cancer cells. Dysregulated expression of checkpoints in the TME is common [41]. *ITGAV* is related to various immune checkpoints in our study and is mainly positively correlated, suggesting its potential for use in immunotherapy.

Some limitations of this study should be noted: (1) there were a lack of in-house samples for exploring *ITGAV* protein levels in pan-cancer; (2) no pure body fluid samples were used to verify the ability of *ITGAV* to screen cancer; (3) limited clinical parameter data were collected in this study, resulting lack of research on the association of *ITGAV* expression with patient backgrounds, such as gender, cigarette consumption, and treatment including immunotherapy; and (4) the complicated molecular mechanisms of *ITGAV* on cancers (such as immune infiltration levels) still need experimental verification in future research.

## Conclusion

In summary, diverse *ITGAV* expression (mainly upregulated) in multiple cancers, its clinical significance, and its potential molecular mechanisms in dozens of cancers were identified in the study. *ITGAV* expression serves as a risk factor for patient prognosis and has distinct effects on patients with some cancers. *ITGAV* may play a role in cancers by participating in immunity-related signaling pathways and influencing the infiltration levels of several immune cells. This study provided valuable insights for a better understanding of the pathogenesis mechanism of *ITGAV* in cancers. *ITGAV* may serve as a potential marker for cancer prognosis and identification and may be associated with immunity.

## Supplementary Information

**Additional file 1.** Cancer abbreviations and full names contained in the pan-cancer dataset in this study.

**Additional file 2.** All data for pan-cancer analyses in this study.

**Additional file 3.** The processes of selecting datasets for this study.

**Additional file 4.** Information of datasets included in the study.

**Additional file 5.** Different smoking histories in SCLC patients with high-*ITGAV* expression and low-*ITGAV* expression. **p of Wilcoxon ran-sum test < 0.01.

**Additional file 6.** There is no significant difference in clinical parameters between high-ITGAV and low-ITGAV expression groups (for disease-free survival).

**Additional file 7.** No *ITGAV* expression levels were observed in SCLC patients with various clinical parameters. *p* value was calculated by Wilcoxon ran-sum tests.

**Additional file 8.** ITGAV expression shows significantly distinctive effects between cancers and non-cancers.

## Data Availability

Datasets from public databases can be obtained from SangerBox (http://vip.sangerbox.com/), ArrayExpress (https://www.ebi.ac.uk/arrayexpress/), GEO (https://www.ncbi.nlm.nih.gov/gds/?term =), GTE-x (https://www.gtexportal.org/home/index.html), and the TCGA Research Network (www.cancer.gov/tcga) with dataset IDs (e.g., GSE32036). Data contained in in-house tissues can be obtained from the corresponding author.
