# Supplementary material for: The clinical significance of integrin subunit alpha V in cancers: from small cell lung carcinoma to pan-cancer
Source: BMC Pulm Med. 2022 Aug 4;22:300. doi: 10.1186/s12890-022-02095-8 (PMC9354352; doi:10.1186/s12890-022-02095-8)
Supplement: Supplementary file 1 — Additional file 1. Cancer abbreviations and full names contained in the pan-cancer dataset in this study. [file 12890_2022_2095_MOESM1_ESM.docx]

**Additional file 1.** Cancer abbreviations and full names contained in the pan-cancer dataset in this study.

| Abbreviation | Full name |
| --- | --- |
| ACC | Adrenocortical Carcinoma |
| BLCA | Bladder Urothelial Carcinoma |
| BRCA | Breast Invasive Carcinoma |
| CESC | Cervical Squamous Cell Carcinoma and Endocervical Adenocarcinoma |
| CHOL | Cholangiocarcinoma |
| COAD | Colon Adenocarcinoma |
| COADREAD | Colon Adenocarcinoma/Rectum Adenocarcinoma Esophageal Carcinoma |
| DLBC | Lymphoid Neoplasm Diffuse Large B-Cell Lymphoma |
| ESCA | Esophageal Carcinoma |
| FPPP | FFPE Pilot Phase II |
| GBM | Glioblastoma Multiforme |
| GBMLGG | Glioma |
| HNSC | Head and Neck Squamous Cell Carcinoma |
| KICH | Kidney Chromophobe |
| KIPAN | Pan-Kidney Cohort (KICH+KIRC+KIRP) |
| KIRC | Kidney Renal Clear Cell Carcinoma |
| KIRP | Kidney Renal Papillary Cell Carcinoma |
| LAML | Acute Myeloid Leukemia |
| LGG | Brain Lower Grade Glioma |
| LIHC | Liver Hepatocellular Carcinoma |
| LUAD | Lung Adenocarcinoma |
| LUSC | Lung Squamous Cell Carcinoma |
| MESO | Mesothelioma |
| OV | Ovarian Serous Cystadenocarcinoma |
| PAAD | Pancreatic Adenocarcinoma |
| PCPG | Pheochromocytoma And Paraganglioma |
| PRAD | Prostate Adenocarcinoma |
| READ | Rectum Adenocarcinoma |
| SARC | Sarcoma |
| STAD | Stomach Adenocarcinoma |
| SKCM | Skin Cutaneous Melanoma |
| STES | Stomach and Esophageal Carcinoma |
| TGCT | Testicular Germ Cell Tumors |
| THCA | Thyroid Carcinoma |
| THYM | Thymoma |
| UCEC | Uterine Corpus Endometrial Carcinoma |
| UCS | Uterine Carcinosarcoma |
| UVM | Uveal Melanoma |
| WT | High-Risk Wilms Tumor |
