# Supplementary figures and images for: The clinical significance of integrin subunit alpha V in cancers: from small cell lung carcinoma to pan-cancer
Source: BMC Pulm Med. 2022 Aug 4;22:300. doi: 10.1186/s12890-022-02095-8 (PMC9354352; doi:10.1186/s12890-022-02095-8)

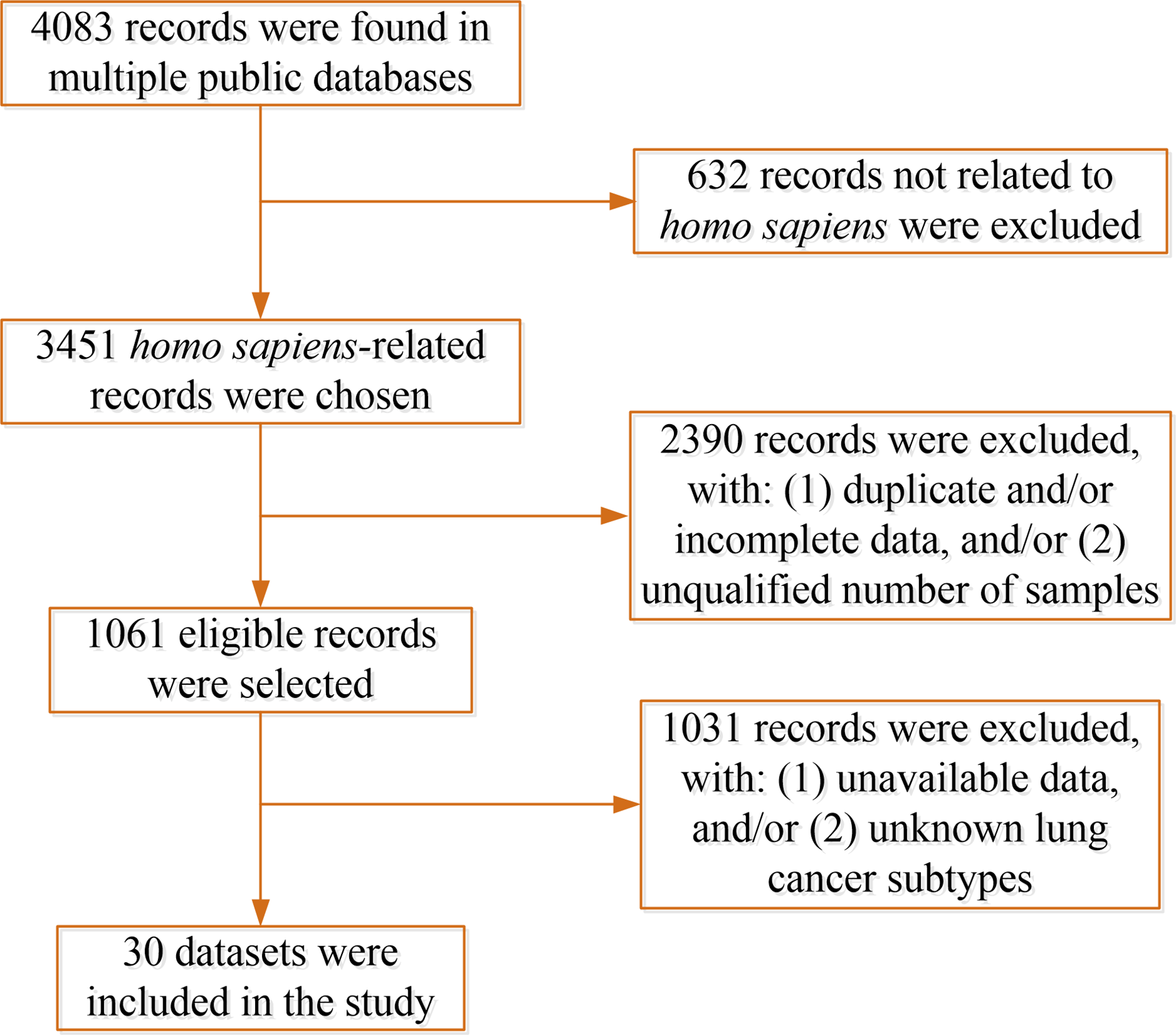

Supplement: Supplementary file 3 — Additional file 3. The processes of selecting datasets for this study. [file 12890_2022_2095_MOESM3_ESM.tif]

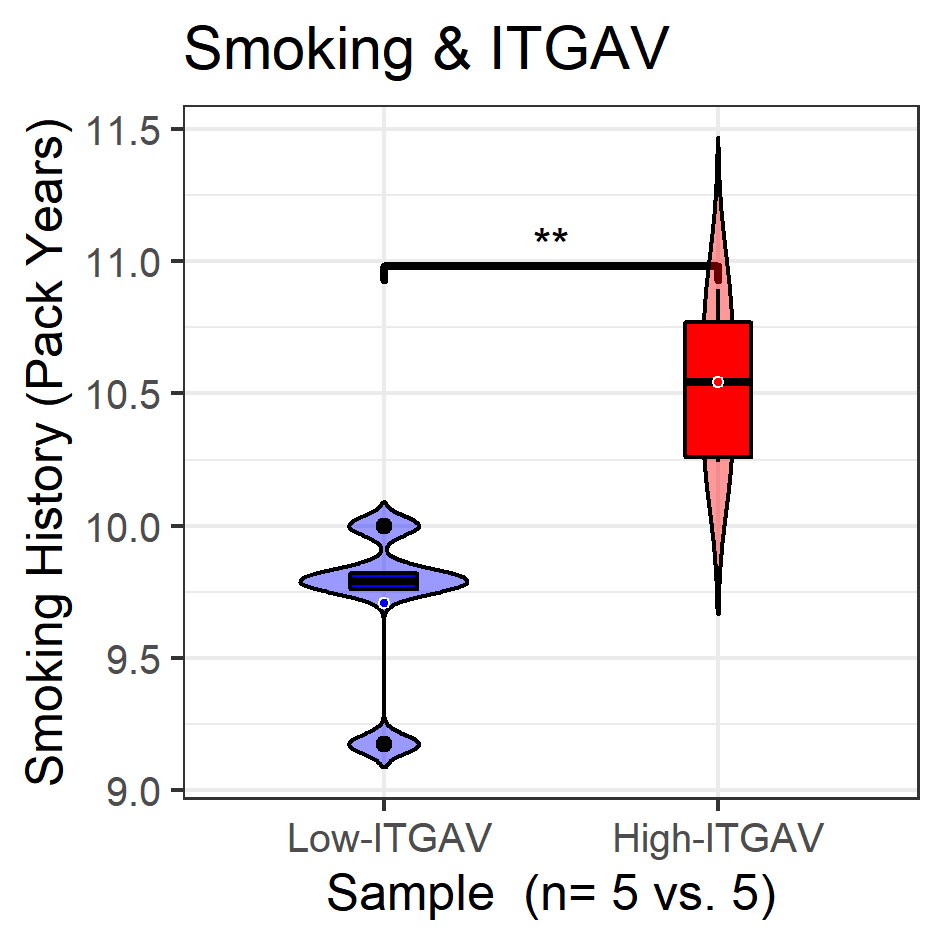

Supplement: Supplementary file 5 — Additional file 5. Different smoking histories in SCLC patients with high-ITGAV expression and low-ITGAV expression. **p of Wilcoxon ran-sum test < 0.01. [file 12890_2022_2095_MOESM5_ESM.tiff]

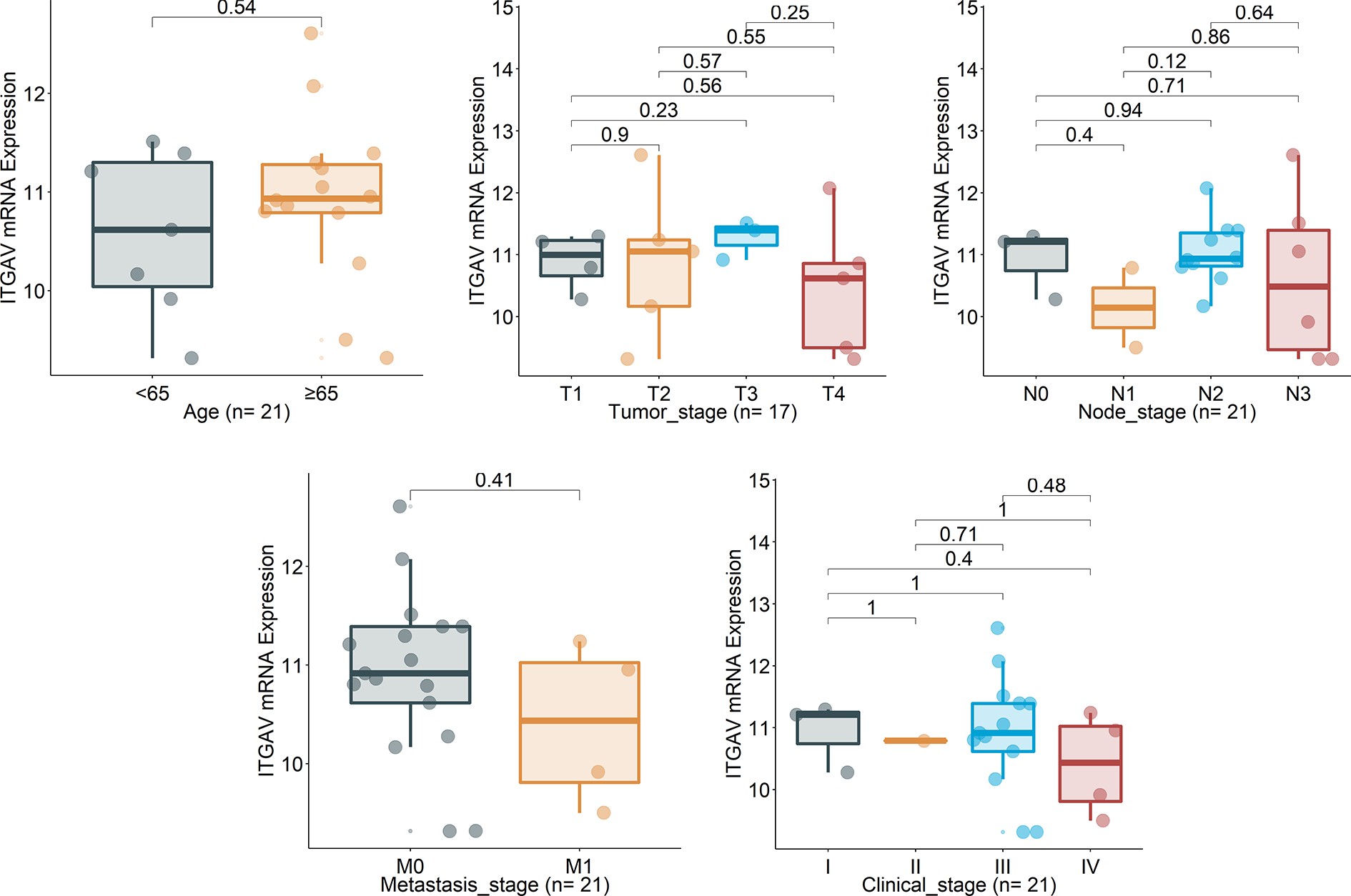

Supplement: Supplementary file 7 — Additional file 7. No ITGAV expression levels were observed in SCLC patients with various clinical parameters. p value was calculated by Wilcoxon ran-sum tests. [file 12890_2022_2095_MOESM7_ESM.tif]

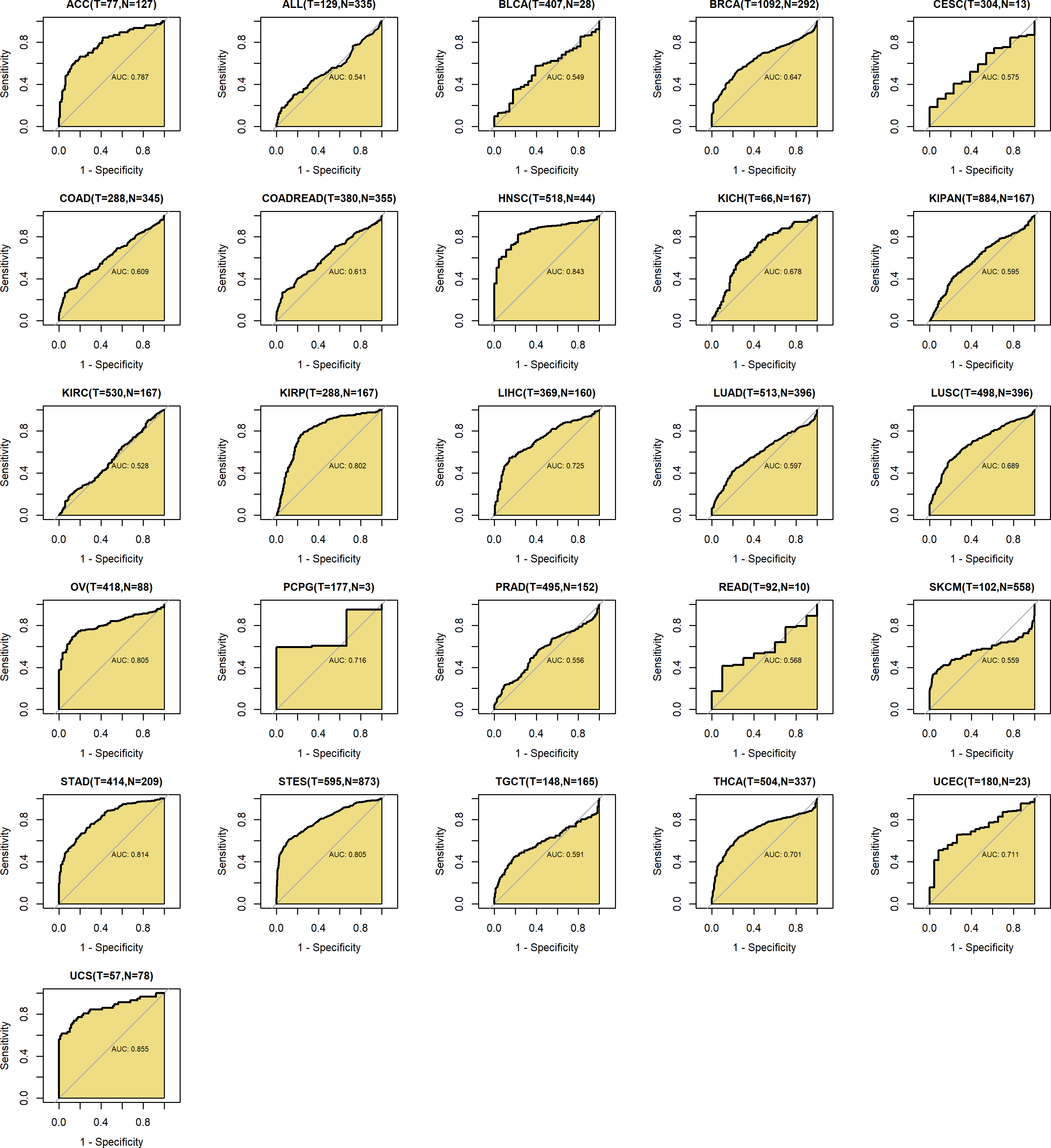

Supplement: Supplementary file 8 — Additional file 8. ITGAV expression shows significantly distinctive effects between cancers and non-cancers. [file 12890_2022_2095_MOESM8_ESM.tiff]
