# Supplementary material for: The clinical significance of integrin subunit alpha V in cancers: from small cell lung carcinoma to pan-cancer
Source: BMC Pulm Med. 2022 Aug 4;22:300. doi: 10.1186/s12890-022-02095-8 (PMC9354352; doi:10.1186/s12890-022-02095-8)
Supplement: Supplementary file 4 — Additional file 4. Information of datasets included in the study. [file 12890_2022_2095_MOESM4_ESM.docx]

**Additional file 4.** Information of datasets included in the study.

| Dataset | Platform | *n* of SCLC^a^ | *n* of non-SCLC | Dataset | Platform | *n* of SCLC | *n* of non-SCLC |
| --- | --- | --- | --- | --- | --- | --- | --- |
| GSE15240 | GPL570 | 23 | 1 | GSE4824-GPL96 | GPL96 | 22 | 8 |
| GSE43346 |  | 38 | 1 | GSE4127 |  | 10 | 0 |
| GSE30219 |  | 21 | 14 | GSE60052 | GPL11154 | 79 | 7 |
| GSE18385 |  | 0 | 31 | GSE83492 |  | 0 | 18 |
| GSE52237 |  | 0 | 37 | GSE4824-GPL97 | GPL97 | 22 | 8 |
| GSE66294 |  | 31 | 0 | GSE6044 | GPL201 | 9 | 5 |
| GSE43079 |  | 0 | 36 | GSE83227 | GPL8300 | 6 | 17 |
| GSE47436 |  | 2 | 0 | GSE108055 | GPL13376 | 12 | 10 |
| GSE45626 |  | 3 | 0 | GSE44447 | GPL14550 | 4 | 7 |
| GSE18674 |  | 0 | 1 | GSE40275 | GPL15974 | 15 | 43 |
| GSE11784 |  | 0 | 125 | GSE1037 | GPL962 | 10 | 18 |
| GSE7832 |  | 0 | 1 | GSE11969 | GPL7015 | 9 | 5 |
| GSE10006 |  | 0 | 60 | GSE149507 | GPL23270 | 18 | 18 |
| GSE8545 |  | 0 | 2 | In-hosue TMA | / | 26 | 29 |
| GSE32036 | GPL6884 | 29 | 59 | Total | / | 455 | 567 |

Notes: ^a^, small cell lung carcinoma; ^b^, tissues microarrays.
