# Supplementary material for: The clinical significance of integrin subunit alpha V in cancers: from small cell lung carcinoma to pan-cancer
Source: BMC Pulm Med. 2022 Aug 4;22:300. doi: 10.1186/s12890-022-02095-8 (PMC9354352; doi:10.1186/s12890-022-02095-8)
Supplement: Supplementary file 6 — Additional file 6. There is no significant difference in clinical parameters between high-ITGAV and low-ITGAV expression groups (for disease-free survival). [file 12890_2022_2095_MOESM6_ESM.docx]

**Additional file 6.** There is no significant difference in clinical parameters between high- ITGAV and low-ITGAV expression groups (for disease-free survival).

| Category | | ITGAV Expression | | *P*-value^a^ |
| --- | --- | --- | --- | --- |
|  |  | High | Low |  |
| Age (year) | <65 | 2 | 5 | 1.000 |
|  | ≥65 | 3 | 9 |  |
| Tumor stage | NA^b^ | 1 | 3 |  |
|  | T1 | 0 | 4 | 0.141 |
|  | T2 | 1 | 4 |  |
|  | T3 | 2 | 0 |  |
|  | T4 | 1 | 3 |  |
| Node stage | N0 | 0 | 3 | 0.731 |
|  | N1 | 0 | 1 |  |
|  | N2 | 3 | 6 |  |
|  | N3 | 2 | 4 |  |
| Metastasis stage | M0 | 5 | 11 | 0.530 |
|  | M1 | 0 | 3 |  |
| Clinical stage | I | 0 | 3 | 0.574 |
|  | II | 0 | 1 |  |
|  | III | 5 | 7 |  |
|  | IV | 0 | 3 |  |

Notes: ^a^P value was calculated based on Fisher’s exact test. ^b^Not applicable.
